# Breast cancer patient-reported outcome of factors influencing cosmetic satisfaction after breast-conserving therapy

**DOI:** 10.1007/s12282-021-01287-0

**Published:** 2021-08-26

**Authors:** A. T. P. M. Brands-Appeldoorn, A. J. G. Maaskant-Braat, L. Janssen, L. A. D. M. van Osch, V. C. G. Tjan-Heijnen, R. M. H. Roumen

**Affiliations:** 1 Department of Surgery, Máxima Medical Centre, De Run 4600, 5500 MB Veldhoven, The Netherlands; 2 Department of Health Promotion, CAPHRI School for Public Health and Primary Care, Maastricht University, PO Box 616, 6200 MD Maastricht, The Netherlands; 3 Department of Clinical Genetics, GROW School for Oncology and Developmental Biology, Maastricht University Medical Centre+, Maastricht, The Netherlands; 4 Div. Medical Oncology, GROW – School for Oncology and Developmental Biology, Maastricht University Medical Centre+, Maastricht, The Netherlands

**Keywords:** Breast-conserving therapy, Breast cancer, Cosmetic outcome, Self-assessment, Body image

## Abstract

**Background:**

The aim of this study was to investigate which factors patients considered to be important for determining the degree of cosmetic satisfaction with regards to perceived body image after previous breast-conserving therapy (BCT).

**Methods:**

Outcomes considered relevant by the patients were first identified using interviews. A questionnaire based on this group input was then devised and added to the physician-based Sneeuw questionnaire. Next, a quantitative study using this questionnaire was conducted in Dutch patients treated at least 6 months earlier for (non-) invasive breast cancer by BCT. Exclusion criteria were: previous mastectomy or BCT of the contralateral breast, BCT with nipple resection, metastatic disease, local recurrence or (previous) plastic breast surgery. Descriptive statistics were used throughout.

**Results:**

A total of 149 patients (aged 36–87 years) completed the questionnaire. From this focus group input, the top three factors in overall importance (important or very important) for satisfaction were: ‘wearability of bra’ (67%), ‘breast sensitivity’ (59%) and ‘asymmetry’ (51%). Younger patients (< 55 years) considered ‘breast size’ to be most important, whereas ‘wearability of bra’ was most frequently reported by older patients (> 55 years). Time since BCT did not significantly influence the rating of relevant factors.

**Conclusion:**

Patients consider ‘wearability of bra’, ‘breast sensitivity’ and ‘asymmetry’ as the most important factors when assessing their satisfaction with regards to cosmetic outcome and body image. These factors should be addressed in routine clinical practice during (pre) counseling.

**Supplementary Information:**

The online version contains supplementary material available at 10.1007/s12282-021-01287-0.

## Introduction

When assessed in terms of local control and survival, the results of breast-conserving therapy (BCT) are at least as good as those of mastectomy in breast cancer patients [1, 2]. An additional goal of BCT is to achieve the best possible cosmetic outcome [3]. Unfortunately, deformation of the treated breast may occur immediately or in the years after treatment.

Although previous studies have described various patient-, tumor- and treatment-related factors that can influence cosmetic outcome following BCT [4–9], the outcome remains difficult to predict. Esthetic and functional outcomes are clearly important issues for patient’s quality of life after BCT [10, 11]. Moreover, it is important to prepare patients for any possible late effects of cancer treatment including the cosmetic outcome. In this way, patients can be counseled as much as possible about what to expect from treatment, so that these issues can be taken into account for their choice of treatment.

Continuous evaluation of cosmetic results is important in view of the ongoing surgical and radiotherapeutic developments in BCT and the increasing use of oncoplastic surgery. Various methods have been described in literature for assessing cosmetic outcome following BCT, both by health professionals and the patients themselves. Cosmetic outcome can be assessed using subjective tools and objective scoring systems [12, 13]. However, cosmetic outcome can also be assessed from the patient’s perspective of their self- and body image. A widely used and validated scale for assessing cosmetic outcome is the Harvard scale, which categorizes a patient’s cosmesis into excellent, good, fair or poor [14]. Another more detailed scale used to rate cosmesis is the questionnaire by Sneeuw et al. [15]. This questionnaire addresses the aspects of: surgical scar, breast size, shape, firmness, color and nipple position. However, this tool was developed by clinicians and without patient involvement: thus it may not contain all of the factors considered important by the patients themselves.

The primary aim of this study was, therefore, to identify factors considered important by the patient’s own assessment of cosmetic outcome and body image following BCT in addition to those evaluated by the Sneeuw questionnaire. We hypothesized that additional factors might play a significant role in determining cosmetic satisfaction and, consequently, on patient quality of life.

Second, we wanted to identify the factors that patients ranked as most important and whether this ranking was dependent on patient’ age and the time since BCT.

## Materials and methods

We performed a quantitative study to identify factors considered important by patients when assessing cosmetic outcome after BCT and their satisfaction with the treated breast.

Participants were recruited from the breast clinic of Máxima MC (MMC), a teaching hospital in Veldhoven and Eindhoven, the Netherlands. Approximately 300 new patients with invasive breast cancer are seen annually in this breast clinic by certified breast surgeons and nurse practitioners.

### Ethics approval

The Medical Ethics Review board of MMC (no. N19.118) determined this study did not require formal ethics approval according to Dutch law (WMO). Data were collected after obtaining informed consent from patients in line with the Declaration of Helsinki [16].

### Interviews with patients

Interviews were first conducted with patients treated by BCT at the MMC. During interview, patients were asked which additional items they considered important in assessing cosmetic outcome and satisfaction, besides those contained in the Sneeuw questionnaire. A nurse practitioner performed these interviews. Data saturation was assumed when a total of 10 patients were interviewed. Their responses were processed into a questionnaire for the present study. The additional items identified as important with respect to cosmetic outcome and body image were: wearability of bra, breast sensitivity, cleavage, asymmetry, altered feeling of the treated breast, sexuality, sports and sauna visit. The difference between the issues ‘breast sensitivity’ and ‘altered feeling’ was discussed with patients as follows: ‘breast sensitivity’ concerns in some way to pain perception, while ‘altered feeling’ refers to decreased sensation of the nipple or skin of the breast. Both issues may have an effect on the perception of sexuality.

### Patients

Subsequently, patients (all Caucasians) treated for invasive breast cancer or Ductal Carcinoma In Situ (DCIS) by BCT at the MMC breast clinic and who had a follow-up visit between December 2019 and March 2020 were included in the study. The follow-up visit had to be at least 6 months after the initial treatment to ensure that radiotherapy treatment was complete.

Excluded were patients with prior mastectomy or BCT of the contralateral breast, BCT with resection of the nipple, metastatic disease at presentation or during follow-up, local recurrence, or any (previous) plastic breast surgery. During the follow-up visit, patients were asked by the surgeon or nurse practitioner to participate in the study and were given further information. After obtaining written informed consent, patients were asked to complete the questionnaire immediately after the follow-up visit to the hospital.

### Patient questionnaire

The questionnaire consisted of 16 multiple-choice questions and one open question (Appendix 1). The first 7 multiple-choice questions were from the original Sneeuw questionnaire [13]. The next 5 multiple-choice questions were based on issues identified during the patient interviews, i.e., ‘wearabilty of bra’, ‘breast sensitivity’, ‘cleavage’, ‘asymmetry’ and ‘altered feeling’. In question 13 (Q13), patients were asked to identify the three most important items for cosmetic satisfaction. In questions 14–16, patients were asked whether the appearance of the treated breast played a role in activities such as sports, sauna visits and sexuality. The final question (Q17) was an open question that gave patients the opportunity to add factors deemed important in relation to cosmetic satisfaction following BCT.

The answer options for the multiple-choice questions consisted of a 5-point scale ranging from "very important” (1) to “unimportant” (5), with a 6th option for “not applicable”.

### Statistical analysis

Data were analyzed using the Statistical Package for the Social Sciences (SPSS, SPSS Inc. Chicago, IL, US), version 22. Descriptive statistics (frequencies and percentages) were used to present results for the answers to the multiple-choice questions. The response to Q13, which ranked the top three factors, was analyzed by calculating sum scores as follows: three points for the highest ranked factor, two points for the second ranked factor and one point for the third ranked factor. The factor with the highest overall sum score was considered the most important overall.

The answers to the multiple-choice questions were dichotomized into: “very important/important” (= important) and “quite important/some importance/unimportant” (= unimportant). Factors that were most frequently selected as ‘important’ were shown for the total group, as well as for subgroups of varying ‘age’ and ‘time since BCT’. The variable 'age' was categorized as follows: ≤ 55, 56–65, 66–75 and > 75 years, while the variable ‘time since BCT’ was categorized as: < 1, 1–2, 2–5, 5–10 and > 10 years.

## Results

Overall, 159 patients received the questionnaire and of these 149 (92%) responded. Of the ten patients excluded, three patients indicated they did not want to fill in the questionnaire for various reasons (‘too tired’ and ‘too confrontational with having had BCT for breast cancer’), while 7 others did not complete the questionnaire in full. The median age of participating patients was 65 years (range 36–87). Table 1 shows the time since BCT. Some patients had BCT 2–5 years ago (28%), 20% of the patients had BCT < 1 year and 15% had BCT > 10 years ago.

**Table 1:** Time since breast-conserving therapy

Time since operation	*N* (%)
< 1 year	30 (20)
1–2 year(s)	23 (15)
2–5 years	42 (28)
5–10 years	32 (22)
> 10 years	22 (15)
Total	149 (100)

### Multiple-choice questions

**Fig. 1:**
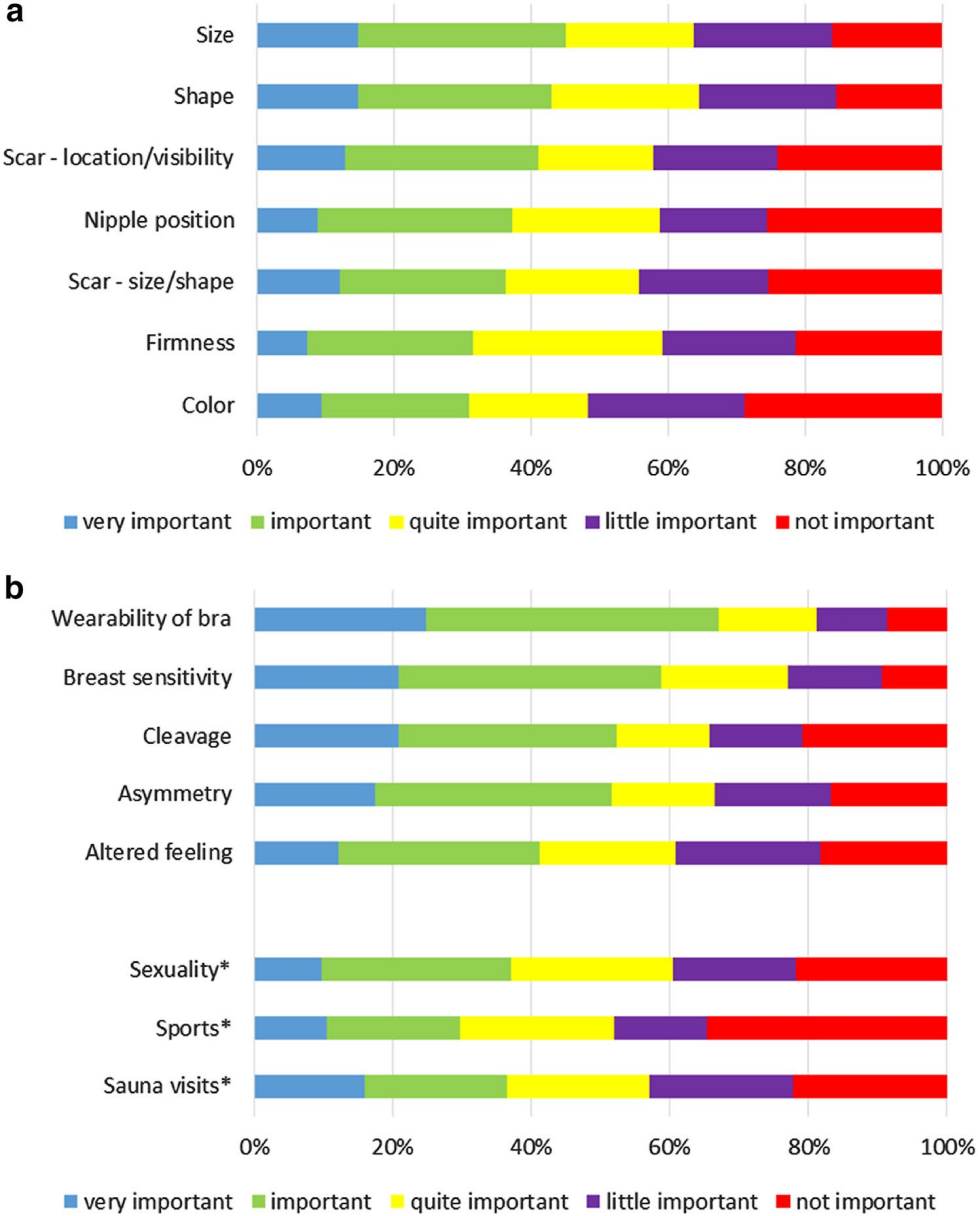
Scores of items from the Sneeuw questionnaire (**a**) and additional factors of the present study (**b**) and outcome of importance. *For the items Sexuality, Sports and Sauna visits respectively 17% (*n* = 25), 30% (*n* = 45) and 58% (*n* = 86) of the patients indicated that this factor was not applicable

Of the 7 items in the Sneeuw questionnaire, size (*n* = 22, 15%) and shape (*n* = 22, 15%) were the factors most often considered as ‘very important’ (Fig. 1a). Figure 1b shows the scores for the 5 additional factors identified during patient interviews. Of these, patients selected 'wearability of bra' as the top factor, with 25% stating it was very important and 42% stating it was important. ‘Breast sensitivity’ was the second highest ranked factor, with 21% stating it was very important and 38% stating it was important.

**Fig. 2:**
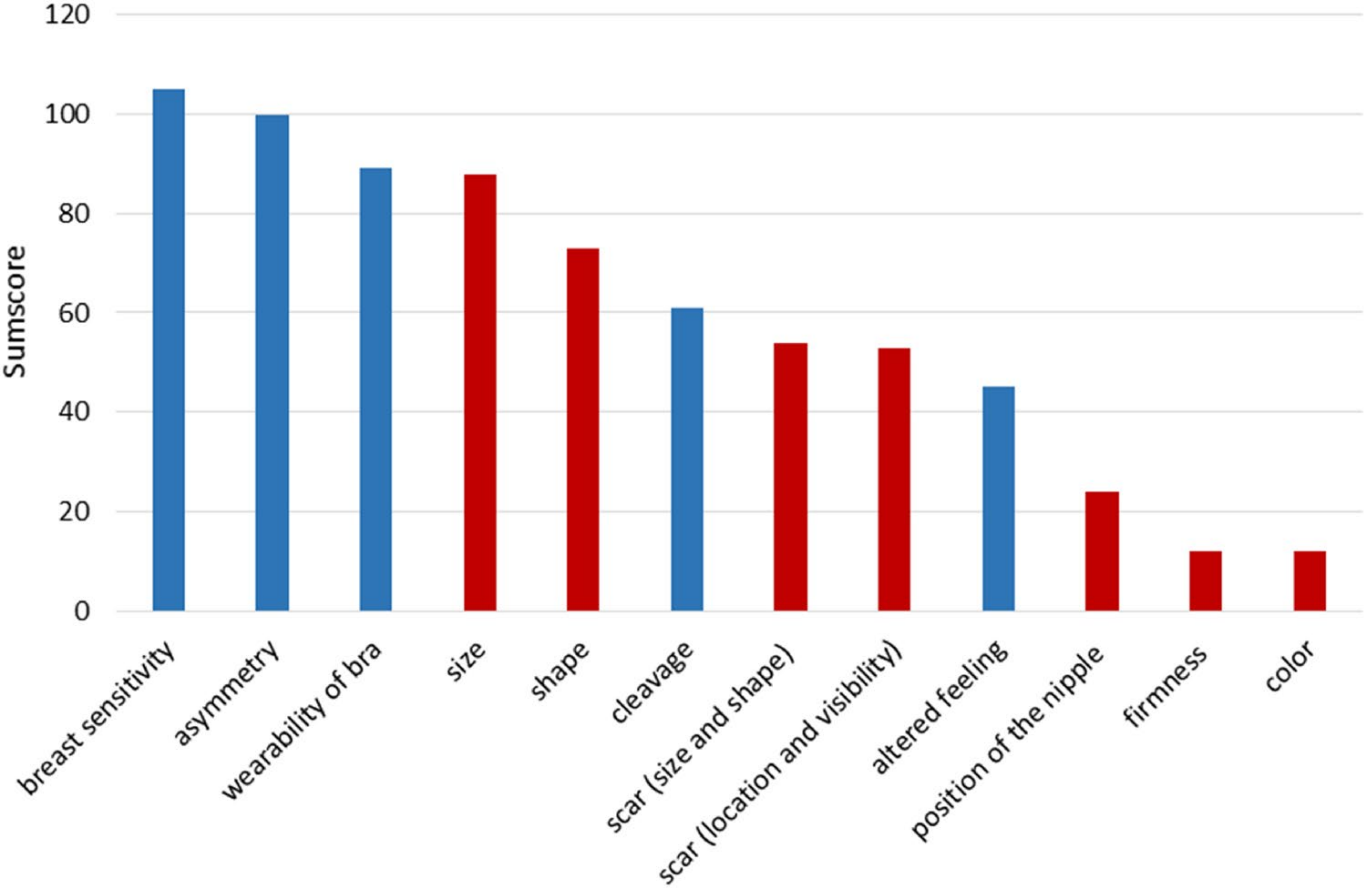
Sumscores for each item, indicating overall importance for the assessment of satisfaction with cosmetic outcome of the breasts. The sumscores are calculated by adding up the points for each patient (3 points for highest ranked item, 2 points for second ranked item and 1 point for third ranked item). A higher sumscore indicates higher importance. The items of the Sneeuw questionnaire are marked in red

Based on the sum scores for the three highest ranking of the 12 factors investigated, patients reported ‘sensitivity of the breast’ to be the most important factor, followed by ‘asymmetry’ and ‘wearability of bra’ (Fig. 2). Table 2 shows that ‘wearability of bra’ was the factor rated most often as ‘very important’ or ‘important’ in the overall group. However, the result for younger women (≤ 55 years) was different to that of older women, with the factors of ‘breast size’ and ‘asymmetry’ rated most often as ‘very important’ or ‘important’. ‘Wearability of bra’ was rated as the most important factor regardless of ‘time since BCT’.

**Table 2:** Items most rated (Sneeuw questionnaire and additional items of the present study) by patients as (very) important for their own assessment of cosmetic outcome of their breast(s) after BCT

		Items most rated as (very) important		
	*N*	Most rated	Second most rated	Third most rated
Total group	149	Wearability of bra	Breast sensitivity	Cleavage
Age				
≤ 55	22	Breast size^1^	Asymmetry^1^	Breast sensitivity and altered feeling
56–65	53	Wearability of bra	Breast sensitivity^2^	Cleavage^2^
66–75	56	Wearability of bra	Breast sensitivity	Cleavage
> 75	18	Wearability of bra	Asymmetry	Breast sensitivity
Time since BCT				
< 1 year	30	Wearability of bra	Breast sensitivity	Size and altered feeling
1–2 years	23	Wearability of bra	Asymmetry	Breast sensitivity
2–5 years	42	Wearability of bra	Breast sensitivity	Multiple items^3^
5–10 years	32	Wearability of bra	Breast sensitivity	Cleavage
> 10 years	22	Wearability of bra	Cleavage	Breast sensitivity

^1^Breast size and symmetry were rated equally often as (very) important within age group ≤ 55

^2^Breast sensitivity and cleavage were rated equally often as (very) important within age group 56–65

^3^Shape, asymmetry and cleavage were all third most rated as (very) important within the group 2–5 years since BCT

For the additional questions regarding: ‘sports’, ‘sauna visit’ and ‘sexuality’, the latter two appeared to be the most important factors (Fig. 1b). Thirty-seven percent of patients indicated that the appearance of breasts was ‘very important or important’ for their ‘sauna visits’ and for their ‘sexuality’. For the factors of ‘sexuality’, ‘sports’ and ‘sauna visits’, 17% (*n* = 25), 30% (*n* = 45) and 58% (*n* = 86) of patients, respectively, indicated that the appearance of breasts was not applicable.

### General remarks made in the open question

Seventy-one patients (48%) responded to the open question (Q17) in which they were given the opportunity to add factors deemed important to the cosmetic appearance of breasts. Patients considered that cosmesis after BCT was important and the majority were satisfied with the outcome. As might be expected, patients considered survival to be more important than the appearance of their breasts following BCT. They were especially happy to have recovered from breast cancer and still be alive. A few of the older patients indicated that, given their age, the cosmetic outcome of their breasts was irrelevant. A minority of the patients who were dissatisfied with their cosmetic outcome indicated they had a worsened body image due to the differences between their breasts. Several patients mentioned the possibility of contralateral breast reduction to counter asymmetry of the breasts. Finally, patients indicated that a nice cleavage was important in allowing more freedom of choice in their clothing.

## Discussion

The present study provides insight into the factors considered important by breast cancer patients with regard to cosmetic satisfaction after BCT. Apart from the items contained in the original Sneeuw questionnaire, additional factors identified by patient interviews were ‘breast sensitivity’, ‘asymmetry of breasts’, ‘wearability of bra’, ‘appearance of the cleavage’ and ‘altered feeling’ in the treated breast. Furthermore, many women reported the appearance of their breasts was ‘very important’ or ‘important’ for their sexuality, sports activities and sauna visits. In a cohort of patients who had undergone BCT at least 6 months earlier, the top three factors in terms of overall importance for satisfaction were ‘breast sensitivity’, ‘asymmetry’ and ‘wearability of bra’, in that order. Interestingly, all three factors are not explicitly included in the original Sneeuw questionnaire. Of note, younger patients assigned more importance to different factors than older (≥ 55 years) patients. ‘Breast size’ mattered more to younger patients, while’wearability of bra’ was more important for the elderly. The time elapsed since BCT did not appear to alter the ranking of these factors. In all categories of ‘time since BCT’, ‘wearability of bra’ was considered to be the most important factor.

Apart from subjective tools, such as the Harvard scale [14] and the questionnaire by Sneeuw et al. [13], objective tools have also been reported previously [7, 8, 17–22]. Both, the subjective and objective tools mainly assess the patient-, tumor- and treatment-related factors that negatively influence general cosmesis. Objective tools focus mainly on the technical aspects of BCT consequences, such as nipple height or retraction and fibrosis of the treated breast. Remarkably, existing tools were developed without any apparent participation by patients. The questionnaire by Sneeuw et al. [15], for instance, was developed by surgical oncologists, plastic surgeons and nurse practitioners, without the involvement of patients (personal communication with N. Aaronson, co-author of Sneeuw et al. [15]). This is the major point of difference with the present study, in which the patient’s own perception of cosmesis and satisfaction were evaluated.

In current routine clinical practice, it is increasingly relevant to know what the patients themselves consider to be important for the outcome of their treatment. The so-called “Patient-Reported Outcome Measures (PROMs)”, of which our questionnaire is a typical example, fits very well into the concept of Value-Based Health Care [23]. The value of care is the highest priority and its quality is determined by the patients themselves.

Based on the present findings, what are the factors that play a role in determining a patient's perception of their body image?

Body image is known to be subject to many changes during oncological treatment [24]. Each treatment modality (surgery, radiotherapy, and systemic therapy) can have a negative impact with great variability in the way the patient perceives the effect of treatment on the integrity of their body.

Four factors that affect a breast cancer patient’s body image have been identified: the patients’ pre-disease personality, socioeconomic factors and common knowledge about cancer prior to treatment, the patients’ age, and the sense of control reported during treatment [24–30]. Another factor that affects body image among patients is ‘shared decision-making’. Patients who are more capable of making their own treatment decisions are then better able to deal with its consequences, thus resulting in a positive influence on perceived body image [26].

The four factors described above that affect a breast cancer patient's body image highlight the key influence of personal characteristics. This may partly explain the present findings of important additional factors to those included in the original Sneeuw questionnaire [15]. A patient’s body image and cosmetic satisfaction are likely to be strongly influenced by their personal characteristics. This supports the assessment of each individual’s own perceptions regarding the outcome of BCT. The most important personal perceptions and considerations should also be included in the counseling.

We feel that ‘wearability of bra’ is a composite factor that incorporates various others, such as ‘asymmetry’, ‘size’ and ‘sensitivity’ of the treated breast.

It may also indicate the possibility of dress (dis)comfort, although this is very personal and may partly contribute to body image. This contrasts then with the more technical items, such as nipple height, color, etc. Although the latter factors can be assessed more objectively by professionals and even computer programs, they may be less relevant for the individual patient involved, as suggested by the present findings. Based on this, healthcare professionals might refer the patient to an advisor for a well-fitting bra, partial prosthesis or even for additional plastic surgery.

In summary, the strength of the present study is its patient-centered approach. This study provides an initial evaluation of the factors considered to be important by the patient’s own assessment of cosmetic outcome and body image following BCT.

The study also has some limitations. First, the independence of the study factors in assessing cosmetic outcome after BCT was not investigated. Second, the variables of ‘breast size’ in the Sneeuw questionnaire and ‘asymmetry’ in the present study, could be interpreted as relating to the same issue. Also, it is unclear to what extend the fact that someone is satisfied with one specific item of the questionnaire may influence the opinion about the importance of that same item.

## Conclusion

Although both objective and subjective tools have been described in the literature to assess cosmetic outcome following BCT, these tools only include factors that healthcare professionals consider to be important. However, in daily clinical practice with PROMs and Value-Based Health Care, the patient’s own input is of great importance. The present exploratory study found that patients also have other considerations when judging their own cosmetic outcome. We recommend that future studies on cosmetic outcomes should incorporate the factors identified in the present work. These factors should also be addressed in daily clinical practice during (pre) counseling.

## Supplementary Information

Below is the link to the electronic supplementary material.

Supplementary file1 (DOCX 25 KB)
