# Supplementary material for: Breast cancer patient-reported outcome of factors influencing cosmetic satisfaction after breast-conserving therapy
Source: Breast Cancer. 2021 Aug 26;29(1):114–20. doi: 10.1007/s12282-021-01287-0 (PMC8732835; doi:10.1007/s12282-021-01287-0)
Supplement: Supplementary file 1 — Supplementary file1 (DOCX 25 KB) [file 12282_2021_1287_MOESM1_ESM.docx]

**Appendix 1**

Patient questionnaire (translated from Dutch)

*Question 1–7: items from the Sneeuw questionnaire*

*Question 8–17: factors from patient interviews*

**How long ago did you have had an operation?**

- Less than 1 year ago
- 1 to 2 years ago
- 2 to 5 years ago
- 5 to 10 years ago
- More than 10 years ago

**Please rate the importance of the items below to rate your own satisfaction with the appearance result (“the cosmetic outcome”) of your treated breast. What makes or determines that you are satisfied with the appearance of your breasts after breast-conserving treatment?**

1. **Nipple position (relative to the untreated breast)**
   - Very important
   - Important
   - Fairly important
   - Little important
   - Not important
2. **Color of the skin (relative to the untreated breast)**
   - Very important
   - Important
   - Fairly important
   - Little important
   - Not important
3. **Breast size (relative to the untreated breast)**
   - Very important
   - Important
   - Fairly important
   - Little important
   - Not important
4. **Surgical scar (size and shape)**
   - Very important
   - Important
   - Fairly important
   - Little important
   - Not important
5. **Surgical scar (location and visibility)**
   - Very important
   - Important
   - Fairly important
   - Little important
   - Not important
6. **Firmness of the breast (relative to the untreated breast)**
   - Very important
   - Important
   - Fairly important
   - Little important
   - Not important
7. **Shape of the breast (relative to the untreated breast)**

- Very important
- Important
- Fairly important
- Little important
- Not important

1. **Asymmetry / difference of the breasts**
   - Very important
   - Important
   - Fairly important
   - Little important
   - Not important
2. **Cleavage**
   - Very important
   - Important
   - Fairly important
   - Little important
   - Not important
3. **Altered feeling in the treated breast**
   - Very important
   - Important
   - Fairly important
   - Little important
   - Not important
4. **Wearability of bra**
   - Very important
   - Important
   - Fairly important
   - Little important
   - Not important
5. **Sensitivity of the treated breast**
   - Very important
   - Important
   - Fairly important
   - Little important
   - Not important
6. **We ask you to indicate which three items from the list above you consider the most important, for your own cosmetic judgment (please place them in order of importance: so at number 1 the item that is most important to you, etc.).**

1…………………………………………..

2…………………………………………..

3…………………………………………..

***Please indicate the importance of the appearance of your breasts (after breast-conserving treatment) in relation to the items in the questions below.***

1. **Sauna visit with other sauna guests**
   - Very important
   - Important
   - Fairly important
   - Little important
   - Not important
   - Not applicable
2. **Sporting**
   - Very important
   - Important
   - Fairly important
   - Little important
   - Not important
   - Not applicable
3. **Sexuality**
   - Very important
   - Important
   - Fairly important
   - Little important
   - Not important
   - Not applicable
4. **There is a possibility to add something personal that may contribute in your own assessment regarding the appearance of your breasts:**

**………………………………………………………………………………………………..**

**………………………………………………………………………………………………..**

**Appendix 2**

Results of questionnaire 17 ***There is a possibility to add something personal that may contribute in your own assessment regarding the appearance of your breasts.*** (translated from Dutch)

Not all patients had completed question 17.

**Study number 1:**

“Think of how the whole process of recovery has transpired and how the regained confidence (that the breast tumor or possibly metastases do not occur) can also influences the satisfaction of the appearance of the breasts after surgery (preferably a scar and a little disfiguration then a relapse)”.

**Study number 2:**

“Survival is more important to me than the appearance of my breasts”.

**Study number 4:**

“Given my age, I don't care what my breast looks like. Most importantly, I'm cured!”

**Study number 8:**

“In retrospect I would have done it differently: at the time I was not aware that an irradiated breast could not endure hardly any cosmetic treatment. If I had known this at the time, I would have had a cosmetic procedure done immediately during the breast-conserving operation. I regret that”.

**Study number 9:**

“I am very satisfied with the appearance of my treated breast!”

**Study number 11:**

“I didn't like my breasts that much anyway, but they have not become uglier as a result of the treatments. Cool scar”.

**Study number 12:**

“Very satisfied with the “recovery” of the breast after surgery”.

**Study number 13:**

“The most important thing is that the operation was successful and the tumor is gone”.

**Study number 14:**

“I was very happy that a breast-conserving treatment was possible”.

**Study Number 16:**

“Color difference of the nipple”

**Study number 17:**

“Can the difference between both breasts be camouflaged with the right choice of clothing or not?”

**Study number 18:**

“I am very satisfied”.

**Study number 22:**

“My breasts are acceptable to me and also to my husband”.

**Study number 24:**

“Very happy that breast-conserving treatment was possible”.

**Study number 26:**

“Satisfied with the healing of the breast and how it looks now”.

**Study number 28:**

“November 2013: For me from the first moment of the diagnoses 'Breast cancer', the most important for me was the best oncological surgeon in the Netherlands. Through medical information via my daughters, it was dr. Roumen. A wonderful person, both medically and as a person!! He had given me confidence. I was healed! The appearance of the breast didn't matter to me. I thought my breast looked great! I had nothing to grumble about! Again, many thanks to dr. Roumen and his team!”

**Study number 31:**

“I don't care. I accept that it is what it is and am very thankful that I am still here!”

**Study number 33:**

“My affected breast was operated on nicely. I am very glad that I am not ill anymore. I think that's more important than what my breast looks like, although I am glad that my breasts are still somewhat similar. I had some great news today, so I am very satisfied and happy! That is more important than the cosmetic aspect!”

**Study number 34:**

“Health is essential to me. Cosmetics of minor importance. But I like to stay informed of the developments regarding the aesthetic”.

**Study number 36:**

“I'm glad I still have the breast, so anything that could be disappointing with regard to shape, etc. is unimportant to me. I have never had any pain, at most a “numb” feeling around the area of the scar”.

**Study number 39:**

“I am very satisfied”.

**Study number 45:**

“I had surgery on my right breast. Result: scar on my right breast (dent in breast). Therefore few negative cosmetic effects for me. Red dots on my skin in my armpit because of radiation. I do not have any negative experience following cosmetic outcome. Important is that everything is fine and that I am doing well. Good luck with the research!”

**Study number 46:**

“That's more general. But a very big fear for me was an amputation, given my age and cosmetic aspect. I am very happy and grateful that breast-conserving treatment was possible for me. The operation is also very good and nicely done. All of this has certainly contributed positively to my recovery (both physically and mentally)”.

**Study number 48:**

“I am very satisfied. Given my age, I can live with the fact that there are minimal differences. That's why I think the wearability of the bra is the most important issue for me. And …let life go on!!!”

**Study number 56:**

“Surgical scar that is getting uglier and more sensitive making an obscuring bra more desirable and harder to find”.

**Study number 58:**

“Well satisfied”.

**Study number 59:**

“I think it's important that the cancer is gone!”

**Study number 60:**

“Operation was very successful, so quite difficult to answer questions critically, because my experience is positive”.

**Study number 62:**

“In general, I am satisfied”.

**Study number 63:**

“I don't feel comfortable about my different breasts, not a good self-image”.

**Study number 65:**

“Although my operated breast looks horrible, it does not hinder me in daily life. I am very happy that I had breast-conserving surgery because I can hide it completely with the help of a good bra. I have had surgery / a lot of tissue removed / blue from the radiation. But nobody sees this/even in a good bathing suit I can hide it. So despite the fact that it is not beautiful, I am very satisfied and even happy with half of my breast”.

**Study number 67:**

“I am 81 years old”.

**Study number 69:**

“What important for me is about the appearance, is that the scar is not just visible, such as with a T-shirt or bathing suit. And a beautiful evening dress”.

**Study number 70:**

“I am very satisfied with my operated breast. Shape-scar satisfied with everything”.

**Study number 71:**

“I am satisfied with the result of my operation and I have barely a visible scar. So many of the questions don't really apply to me!”

**Study number 77:**

“Hyperbaric oxygen therapy has helped me a lot in terms of scar and color. Burnt - due to irradiation, the skin looked very dark and ugly at the time”.

**Study number 79:**

“What the breast looks like when you wear a bra. Some professions require you to change clothes in front of others”.

**Study number 80:**

“Appearance does not matter. Wearing a well-fitting bra is important”.

**Study number 82:**

“Angelique, good luck with the research!”

**Study number 83:**

“I'm still here, that's the most important thing for me”.

**Study number 84:**

“Health is the most important thing to me, the rest is secondary”.

**Study number 87:**

“I find the experience of radiation very disappointing and because of the effects I would not choose the same treatment now! I feel like I did more damage as a result”.

**Study number 89:**

“I am very satisfied with the work of the surgeon who placed the scar just below the nipple. I'm glad I'm doing well and that makes the appearance of the breast much less important to me”.

**Study Number 90:**

“The difference in size and firmness is the only visible thing when I'm just dressed”.

**Study number 92:**

“Doesn't matter. Must be age”.

**Study number 94:**

“Looks good and the aftercare and attention-guidance by Angelique is great. A 10”.

**Study number 95:**

“I am satisfied with the size of the tumor and treatment: seeing that the tumor was situated so deep”.

**Study number 96:**

“I don't wear bras and that means that in a t-shirt or shirt it is always visible that my right nipple is lower than the left, I do not see it by myself anymore, I don't think that's important either, but I do notice that other people notice it. However, that is not so decisive that I dress myself differently or wear a bra. In the sauna I am happy that my scar is mainly under my armpit. I am very happy with the results of my breast-conserving surgery!”

**Study number 97:**

“I am satisfied with the appearance of my breasts”.

**Study number 98:**

“Shape, thickness, location and sensitivity of the scars. Overall shape of the breast. No dents. Nice if there is still some symmetry with the other breast (similar shape, for example: apple-apple, pear-pear).

Good luck with your research”.

**Study number 100:**

“This does not play a role for women older than 70 years”.

**Study number 101:**

“I'm not concerned with that at all, only sensitivity is occasionally disturbing”.

**Study number 103:**

“I have therapy for my breasts. This is necessary. I regret this. For the rest it goes well”.

**Study number 105:**

“The location of the tumor is very decisive. In my situation, the tumor was on the outside, so I have little or no discomfort from the operation and scar. This would be different if the place would have been more towards the middle”.

**Study number 113:**

“I think my appearance is horrible. Of course I had to accept it. My husband had died of cancer (lung) 2 years before the diagnosis (2002). After his death, I never found a relationship that could match him. So I live alone, happy and in good health”.

**Study number 117:**

“I am very happy that I was allowed to keep my breast”.

**Study number 120:**

“Nothing to add”.

**Study Number 122:**

“Gratitude that all is well”.

**Study number 129:**

“I am very satisfied with everything I have undergone”.

**Study number 135:**

“Nothing is “important”. Only healing!”

**Study number 137:**

“I am very satisfied”.

**Study number 139:**

“I'm glad I had survive the disease, now 8 years ago. I can think of everything that could be nicer and better. But I can be very happy that my breast was spared. Dr. Schenk operated very nicely and the scar has remained beautiful. The only bad thing is that my nipple is pulled in. Probably because of the radiation”.

**Study number 146:**

“I'm glad I had a breast reduction in the untreated breast. As a result, a fair/good symmetry again”.

**Study number 147:**

“Pain complaints caused by radiation are very important for satisfaction”.

**Study number 149:**

“Fortunately, I have no extreme changes in the treated breast compared to the situation before. And no extreme changes compared to the untreated breast. So I am extremely satisfied with current situation”.

**Study number 152:**

“Edema in breast and arm”.

**Study number 154:**

“I only care about my health. For me, the aesthetic part of my breast is unimportant”.

**Study number 157:**

“Self-confidence”.

**Study number 159:**

“The breast- conserving operation has reduced the size of the right breast. Given my age, I can live with that”.

**Study number 161-1:**

“I don't mind the way they look. I'm glad I'm well again, so then certain things (for me), like how they look, become a lot less important”.

**Study number 161-2:**

“You still want to look a bit nice and synchronized, so that you can also wear a bra and bathing suit in the normal way”.
